# REST Regulates Distinct Transcriptional Networks in Embryonic and Neural Stem Cells

**DOI:** 10.1371/journal.pbio.0060256

**Published:** 2008-10-28

**Authors:** Rory Johnson, Christina Hui-leng Teh, Galih Kunarso, Kee Yew Wong, Gopalan Srinivasan, Megan L Cooper, Manuela Volta, Sarah Su-ling Chan, Leonard Lipovich, Steven M Pollard, R. Krishna Murthy Karuturi, Chia-lin Wei, Noel J Buckley, Lawrence W Stanton

**Affiliations:** 1 Stem Cell and Developmental Biology, Genome Institute of Singapore, Singapore; 2 Computational and Mathematical Biology, Genome Institute of Singapore, Singapore; 3 Genomic Technology and Biology, Genome Institute of Singapore, Singapore; 4 Centre for the Cellular Basis of Behaviour, Institute of Psychiatry, King's College London, United Kingdom; 5 Department of Biological Sciences, National University of Singapore, Singapore; 6 Wellcome Trust Centre for Stem Cell Research, University of Cambridge, United Kingdom; Baylor College of Medicine, United States of America

## Abstract

The maintenance of pluripotency and specification of cellular lineages during embryonic development are controlled by transcriptional regulatory networks, which coordinate specific sets of genes through both activation and repression. The transcriptional repressor RE1-silencing transcription factor (REST) plays important but distinct regulatory roles in embryonic (ESC) and neural (NSC) stem cells. We investigated how these distinct biological roles are effected at a genomic level. We present integrated, comparative genome- and transcriptome-wide analyses of transcriptional networks governed by REST in mouse ESC and NSC. The REST recruitment profile has dual components: a developmentally independent core that is common to ESC, NSC, and differentiated cells; and a large, ESC-specific set of target genes. In ESC, the REST regulatory network is highly integrated into that of pluripotency factors Oct4-Sox2-Nanog. We propose that an extensive, pluripotency-specific recruitment profile lends REST a key role in the maintenance of the ESC phenotype.

## Author Summary

Embryonic stem cells have the unique and defining property of pluripotency: the ability to differentiate into all cell types. Key transcription factors form interconnected gene regulatory networks that control pluripotency and differentiation. Recently, the transcriptional repressor RE1-silencing transcription factor (REST) was implicated in the maintenance of pluripotency. This was surprising, given that REST has long been known as an essential regulator of neurodevelopment. How does REST regulate pluripotency? Does REST have distinct cohorts of binding sites and target genes in different developmental contexts? To address these questions, we made whole-genome maps of REST binding sites in two mouse stem cell types: embryonic (ESC) and neural (NSC) stem cells. These data were compared with each other and with gene expression data from cells in which REST activity was inhibited. The target genes were almost completely distinct in the two cell types. Surprisingly, we found that REST recruitment has two approximately equal components: common sites across all cells and an ESC-specific component. These pluripotency-associated sites are enriched for particular classes of genes, including those mediating the Wnt signaling pathway, which is an essential regulator of pluripotency.

## Abstract 3

Whole-genome mapping of the essential transcriptional repressor REST reveals distinct binding profiles and diverse roles in embryonic and neural stem cells.

## Introduction

Differentiation of pluripotent embryonic stem cells (ESC) is accompanied by wholesale changes in the transcriptome and epigenome [1–4]. Conversely, an intricate and integrated network of transcriptional regulators is responsible, under the correct conditions, for maintaining ESC in their unique, undifferentiated state. Several key transcription factors required for maintaining pluripotency have been identified and include Oct4, Sox2, and Nanog. The scale of the transcriptional regulation governed by these factors is apparent from recent genome-wide chromatin immunoprecipitation (ChIP) studies, which have identified thousands of genomic binding sites for Oct4 [2,5], Sox2 [2], Nanog [5], and c-Myc [6]. However, ChIP studies reveal only occupancy and cannot, by themselves, indicate the functionality of any bound transcription factor. Nor is it is known how the occupancy and efficacy of any particular transcription factor vary across different cell types. These issues are particularly germane to pluripotent and multipotent stem cells, where expression of individual transcription factors can lead to differentiation and wholesale changes in the cellular transcriptome. For instance, the HMG protein Sox2 is required both for maintenance of pluripotency in ESC [7] and for maintenance of the undifferentiated state in neural progenitors [8,9]. Further evidence for diversity of function can be seen with Oct4: Although primarily associated with pluripotency, forced expression of Oct4 can also promote neurogenesis [10]. In parallel to maintaining the undifferentiated state, there is also a strict requirement for both pluripotent and tissue-specific stem cells to suppress expression of inappropriate lineage-specific genes. In ESC, this is manifested as silencing of all lineage-specific genes, whereas in committed neural stem cells (NSC), precocious expression of differentiated neuronal genes must be prevented. In both cases, neuronal gene expression must be suppressed. One factor that is responsible for this function in both ESC and NSC is REST (RE1-silencing transcription factor).

REST (also called NRSF) is expressed throughout early development where it represses expression of neural genes in both ESC [11] and NSC [12,13]. However, REST appears to have quite different roles in the two cell types. Whereas REST does not appear to be necessary for differentiation of the blastocyst into the three germ layers or for formation of the neural plate and early neural tube [14], down-regulation of REST is required, and in some cases is sufficient, for neuronal differentiation [2,5,11,13,15,16]. The observation that REST is directly regulated by Oct4, Sox2, and Nanog [5] provides an intriguing direct linkage between suppression of neuron-specific genes and pluripotency. A direct interaction between REST and Nanog proteins also links these two regulatory networks [17]. However, it remains unknown whether these cell-specific transcription programs are underwritten by interaction of REST with distinct sets of target genes in ESC and NSC.

Bioinformatic studies [17–20] have identified several thousand potential REST binding sites in the human and mouse genomes, while numerous ChIP studies have shown that REST is present at distinct sites in different cell types [20]. For instance, REST is present at the RE1 site of the *Bdnf* gene in chromatin from mouse forebrain, but cannot be detected at the same locus in mouse liver [21]. However, recent genome-wide ChIP sequencing studies have shown that REST can be detected at most RE1 sites in a human T cell line and a mouse kidney cell line [22,23]. Although the precise reasons for this apparent disparity are not clear, a strong possibility is that the widespread occupancy detected by ChIP-SACO (serial analysis of chromatin occupancy) and ChIP-seq is a reflection of the increased sensitivity of these deep-sequencing–based methodologies and their ability to detect low-level or transient interactions.

In this study, we have taken a three-pronged approach to compare and contrast the REST regulatory network in mouse ESC and NSC. Firstly, we have used an array-based chromatin immunoprecipitation microarray (ChIP-chip) approach to examine differences in target gene occupancy by REST in both ESC and NSC, as well as in differentiated fibroblasts. Secondly, we have used an unbiased, genome-wide approach to identify novel REST binding sites by applying a deep sequencing chromatin immunoprecipitation paired-end tag (ChIP-PET) strategy. Thirdly, we have identified those genes whose transcription is regulated by REST by comparing the transcriptomes of ESC and NSC before and after blocking REST activity with a specific dominant-negative construct. We find that REST binding sites can be classified into cell-type–independent loci, which are bound in all cell types we examined, and an ESC-specific set, which appear to regulate genes involved in signaling and transcriptional regulation related to pluripotency. Inhibition of REST function by overexpression of a dominant-negative construct revealed that the genes regulated by REST in ESC and NSC are almost completely different. Thus, although there is an extensive common core of occupied REST sites in ESC and NSC, the REST regulatory networks operate distinctly in the two cell types. This is, to our knowledge, the first study that has compared the regulatory network of any transcription factor in pluripotent ESC and multipotent NSC, and it lends novel insights to lineage specific regulation of gene expression.

## Results

### A Microarray-Based Approach for Mapping REST Binding Sites across the Genome

To broadly interrogate in vivo the occupancy of the REST transcriptional repressor across the genome of mouse stem cells, a DNA microarray was developed to be used in combination with chromatin immunoprecipitation (ChIP-chip). The microarray was spotted with 1,095 oligonucleotide probes (Dataset S1) representing RE1 sites computationally predicted by the 21-bp RE1 position-specific scoring matrix (PSSM) [18]. A unique probe was designed within a 200-bp window centered on each site, excluding the actual RE1 site to avoid cross-hybridization. The ChIP-chip microarray also included 92 probes from intergenic and coding regions of the genome that are distal from any known RE1, to serve as negative controls. To optimize and monitor performance of our microarray, multiple probes were tiled across RE1 sites associated with the REST target genes *Nppa* [24,25] and *Syt4* [20]. We applied this ChIP-chip methodology to map REST occupancy in genetically identical mouse ESC and NSC.

**Figure 1:**
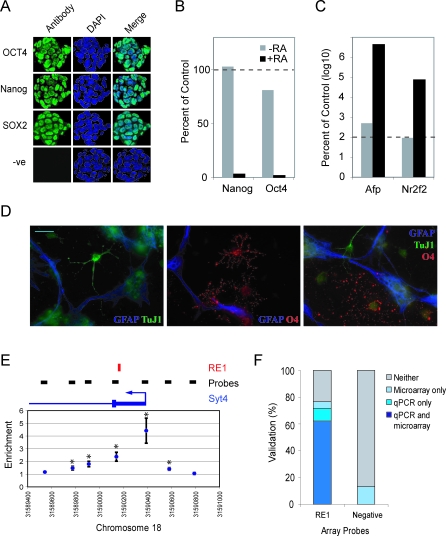
Validation of Stem Cell Potency and Microarray Sensitivity (A) Mouse ESC stain for markers of self-renewal. Immunohistochemistry was carried out using antibodies to Oct4, Nanog, and Sox2. For negative control (-ve), the primary antibody was omitted. (B and C) ESC are capable of differentiation. Following treatment with retinoic acid (RA), qRT-PCR was used to assay expression of self-renewal markers (*Nanog*, *Oct4*) (B) and differentiation markers (Alpha fetoprotein, *Afp*; Coup TFII, *Nr2f2*). (D) NSC are multipotent. Following a standard differentiation protocol (see Text S1), cells were observed to differentiate into neurons, astrocytes and oligodendrocytes, as revealed by staining with Tuj1 (βIII tubulin), GFAP (glial fibrillary acidic protein) and O4 antibodies, respectively. Scale bar: 20 μm. (E) Enrichment for REST occupancy in ESC at the *Syt4* RE1 site as determined by ChIP-chip. Locations of seven tiled probes and RE1 site are indicated. (F) Comparative performance of ChIP-chip and qPCR for 150 randomly selected RE1s. Dark blue (occupied) and gray bars (unoccupied) indicate a consistent call by both techniques. Light blue bars denote RE1s that were not consistently called.

ChIP DNA was isolated from undifferentiated pluripotent ESC (Figure 1A–1C and Figure S1) and multipotent NSC (Figure 1D and Figure S2) and hybridized to the RE1 ChIP-chip. Statistically significant enrichments were determined by a combination of Gaussian Mixture Model and Single Array Error Model [26] analyses. The false discovery rate (FDR) was selected to optimize sensitivity and selectivity, by comparing both statistical and experimental evaluations of the microarray. The enrichments detected over the tiled regions of *Nppa* and *Syt4* were specific and restricted to small regions around the predicted RE1 sites (Figure 1E). To validate the ChIP-chip performance, quantitative PCR (qPCR) was carried out on a randomly selected set of RE1 sites (Figure 1F and Figure S3). Taking qPCR as the standard, the ChIP-chip correctly called REST occupancy, or lack thereof, at 108 out of 126 RE1 sites (86%). Importantly, 87% of negative control probes were correctly called by the ChIP-chip, indicating a false positive rate of around 13% as expected from the statistical model. With the statistical parameters used in our studies, we found that the ChIP-chip method accurately assessed REST occupancy at the 1,095 predicted RE1 sites. The focused REST ChIP-chip thus provides a robust and rigorous means to compare occupancy of REST across many cell types and under various stages of differentiation, and thereby reveals a more complete and dynamic view of the REST transcriptional regulatory network.

### Distinct REST Occupancy Patterns in ESC, NSC, and Fibroblasts

**Figure 2:**
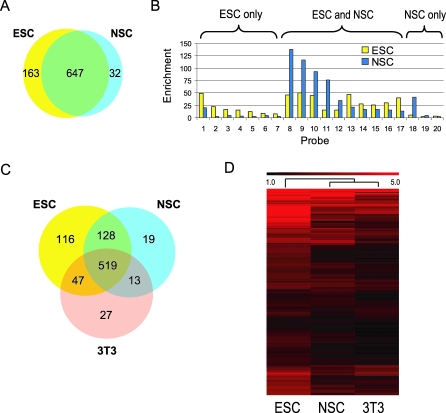
Cell-Type–Specific Recruitment Patterns of REST as Revealed by ChIP-chip (A) The Venn diagram shows the numbers of bound RE1s in ESC (yellow) and NSC (blue) as determined by ChIP-chip. An additional 253 sites were not occupied in either cell type. (B) Representative qPCR validation of ESC-specific, NSC-specific, and shared sites. (C) Comparison of REST-occupied sites in NIH3T3, ESC, and NSC. (D) Hierarchical clustering of 564 commonly occupied RE1 from (C). Red scale reflects relative enrichment at each RE1.

We used RE1 ChIP-chip to compare genome-wide REST occupancy across different cell types. It has been established that REST acts by directly binding and recruiting corepressors to RE1s associated with target genes [27]. It is intriguing that REST is expressed in both lineage-restricted neuronal progenitors [12,14,28] and pluripotent ESC [11,13], given their distinct differentiation potentials. We sought to understand better the role that REST plays in these distinct contexts by applying our ChIP-chip method to compare REST occupancies in these two cell types. The E14 ESC line and the NS5 NSC line [29], which was derived from E14 by in vitro differentiation, provided a genetically matched and developmentally linked pair of stem cell types to compare. One major advantage of the ChIP-chip platform is the ability to perform numerous replicate experiments, quickly and affordably, which thereby provides statistically rigorous results. ChIP material was prepared from five independent biological replicates of both ESC and NSC. These experiments identified 810 and 679 RE1 sites that showed statistically significant binding in ESC and NSC, respectively (Figure 2A). There were 647 sites commonly occupied in both cell types, 163 sites occupied only in ESC, and 32 sites occupied only in NSC. We performed qPCR validation on randomly selected RE1s (Figure 2B). The rate of validation was 8/10 (80%) for commonly bound sites (Figure S4). Seven out of 13 (54%) of ESC-specific RE1s were correctly called by the ChIP-chip (Figure S5). In contrast, we tested all 32 NSC-only sites and found just one that was NSC-specific: the remainder had either detectable recruitment in ESC or no recruitment in NSC (Figure 2B and Figure S6). Statistically, this is not unexpected given the small number of NSC-only sites detected (32 out of 879 total sites or 4.7%), and is consistent with an overall FDR of 10% for the ChIP-chip data. In summary, 60% of all RE1 sites on the ChIP-chip were occupied in both ESC and NSC, approximately 7% were occupied in ESC but not NSC, and there were very few, if any, sites occupied only in NSC. We conclude that the RE1 sites occupied in the NSC are a subset of those occupied in ESC.

We also used the ChIP-chip method to profile REST occupancy in a differentiated cell type, NIH3T3 fibroblasts (3T3) (Figure 2C). A total of 606 REST binding sites were detected; of the 27 RE1s reported to be 3T3-specific, we found none that were validated as such by qPCR (Figure S7). It is interesting that a core set of 519 (44%) RE1 sites was occupied in all three cell types. Hierarchical clustering was performed to group the three cell types based on the overall similarity of their REST recruitment profile (Figure 2D). It is apparent that the occupancy levels at these common sites are unique for each cell type. Overall, the occupancies were greatest in ESC, although many sites displayed the highest degree of occupancy in NSC or 3T3 cells. The clustering determined that the 3T3 and NSC REST binding profiles are more similar to each other than to ESC, suggesting a pluripotency-associated REST recruitment profile. Thus, although there are many REST binding sites commonly occupied, these data reveal a cell-type specific REST occupancy signature.

### Genome-Wide Identification of Novel REST Binding Sites

**Figure 3:**
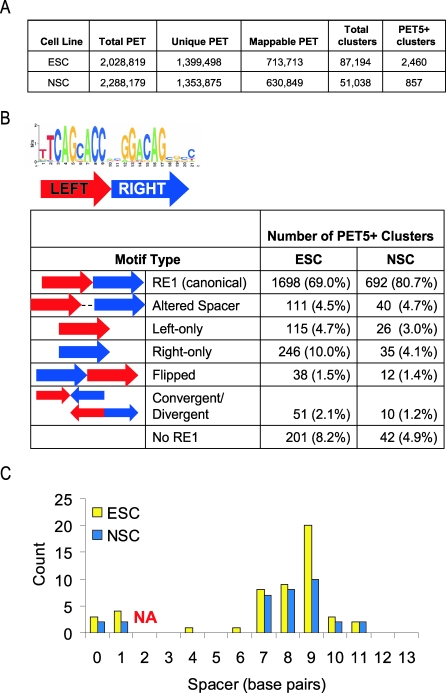
ChIP-PET Identification of Noncanonical REST Binding Motifs (A) Sequencing and mapping statistics for REST ChIP-PET in ESC and NSC. (B) REST binding sites identified by ChIP-PET were classified by similarity to the full-length RE1 motif, and orientation of the left (red) and right (blue) RE1 half-sites. (C) Number of sites with altered spacing between the left and right half sites, compared with the canonical 2-bp spacer. Shown is a histogram of the number of instances for each spacer size, for the PET5+ sites from ESC (yellow) and NSC (blue). NA, not applicable (i.e., canonical 2-bp spacer).

The RE1 ChIP-chip approach described above interrogated high-quality RE1 motifs that had a strong match to the RE1 PSSM. However, recent reports have identified noncanonical RE1-like motifs that can effectively recruit REST in vivo [22,23,30]. To extend our study to the unbiased identification of REST binding sites, we generated a genome-wide map of REST binding in ESC using ChIP-PET technology, which combines ChIP with deep DNA sequencing [31]. Briefly, a library was prepared from ChIP DNA in a manner that produced paired-end tags (PETs) for each DNA fragment. The PETs were sequenced and mapped to the mouse genome to define the chromosomal locations where REST is bound in vivo. This ChIP-PET technique has been used previously to map binding sites for several transcription factors in ESC, including Oct4 and Nanog, and has been shown to be accurate and sensitive [5,31]. ChIP-PET mapping of REST in ESC generated 713,713 nonredundant PETs that clustered (i.e., overlapped at the same genomic location) into 2,460 high-confidence REST binding sites (Figure 3A). A confidence threshold was set at PET clusters of five or more unique and overlapping members (PET5+) based upon ChIP-PCR validation of sets of 20 genomic loci, each randomly selected from clusters of size three to ten (PET3–PET10) (Figure S8). Seventy-five percent (39/52) of PET5–PET7 clusters we tested had detectable REST binding by qPCR. Further evidence for the efficacy of the method is demonstrated by the fact that PET5+ clusters overlap 91% (649/714) of the RE1s identified by ChIP-chip in ESC (Figure S9).

Closer analysis of the 2,460 high-confidence clusters found that 665 of the PET sites corresponded to 716 of the predicted RE1 sites (the numerical differences are due to instances where multiple RE1 sites map within the span of a single PET cluster). Thus, there were 1,795 additional PET clusters that did not map to computationally predicted RE1 sites. The sequences of the 2,460 PET clusters were analyzed for the presence of common motifs that might represent novel REST binding sites, using the Weeder algorithm [32]. Consistent with previous reports [22,23], several classes of DNA sequences related to the known RE1 were discovered in the REST PET sequences (Figure 3B). As expected, many of the binding sites matched closely to the consensus RE1 motif (70%), albeit with a weaker homology than those sequences included on the RE1 ChIP-chip. Other binding sites were composed of two half sites that matched the RE1 consensus, but with altered spacing between the half sites (5%). The distance between the half sites was found most often to be shortened to none or one base pair, or expanded by 7–11 base pairs (Figure 3C). In other instances, a left or right half site was found with no corresponding partner (5% and 10%, respectively), or had the paired half-sites arranged in atypical orientations (5%). Thus, the RE1 motif can accommodate flexible spacing between its half sites (Figure 3B). However, this new consensus RE1 motif does not account for all the REST binding sites that we mapped by ChIP-PET, since 201 (8%) had no resemblance to an RE1 consensus motif or half site. We found no alternative consensus motif among these atypical sites. Nonetheless, these are true REST binding sites as their recruitment of REST was validated by qPCR (Figure S10). This recruitment may be direct, through noncanonical REST-DNA interactions, or indirect through recruitment of REST by other DNA-bound factors.

We also performed ChIP-PET in NSC; this generated 630,849 PETs that cluster into 857 high-confidence REST binding sites (Figure 3A). Unexpectedly, there were about 3-fold fewer high-confidence REST binding sites identified in NSC compared with ESC. This difference cannot be explained by the depth or quality of the sequencing as the total number of sequenced and mapped PETs was similar for the two samples. qPCR validated all of the randomly selected, commonly bound PET clusters we tested (Figure S11). Thus the ChIP-PET method effectively identified REST binding sites in both ESC and NSC. As before, a de novo motif search was carried out on NSC PET clusters, with similar results as for ESC (Figure 3B) (A comprehensive set of all RE1 motifs identified in ESC and NSC PET clusters can be found in Datasets S2 and S3). These results are in accordance with findings from kidney and lymphoblastoma cells [22,23].

**Figure 4:**
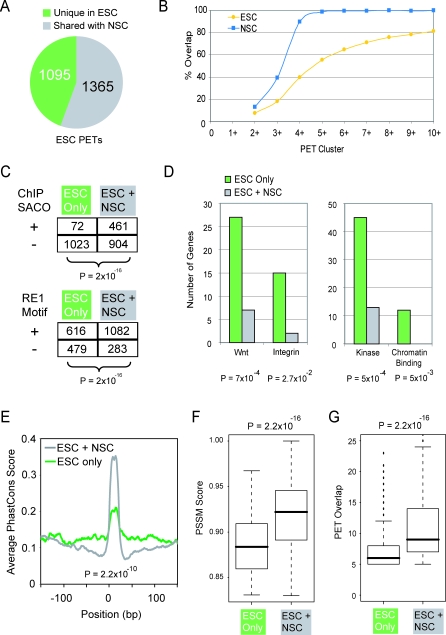
ESC-Specific Gene Targeting by REST (A) ESC-specific REST binding sites (green) are defined as those loci having PET10+ in ESC and no evidence (PET1 or no PET) in NSC. (B) For ESC (yellow) or NSC (blue), the fraction of PET clusters of a given size range that also overlap a PET5+ cluster from the other cell type is plotted. (C) The numbers of ESC-specific and common REST PETs shared with kidney cells (from ChIP-SACO [22]), and the numbers overlapping full-length RE1 motifs are shown. Statistical significance was calculated using the Chi-squared test. (D) Genes involved in Wnt and integrin signaling, and genes encoding kinases and chromatin-binding proteins are enriched amongst targets of ESC-specific REST PET clusters. Shown are the numbers of genes and *p*-value (Chi-square test) for each ontology term among annotated targets of the PET clusters from (A). (E) ESC-specific and ESC-NSC common binding sites have distinct sequence conservation. All REST PET clusters with an identifiable, consensus RE1 were aligned in the same strand orientation. The mean PhastCons sequence conservation score [54] for every position in a 300-bp window around the RE1 is plotted. Statistical significance is based on comparing the mean Phastcons score across every 21mer RE1 (Student's *t-*test). (F and G) Boxplots show the distribution of (F) RE1 PSSM scores [18] and (G) PET overlap count for ESC-only and common ESC-NSC PET clusters. Statistical significance was calculated using Student's *t*-test.

REST binding profiles in ESC and NSC, as determined by ChIP-PET, were compared to discover cell-type specific binding sites. Of the 2,460 high-confidence sites (PET5+) found in ESC, 1,365 were also identified in NSC. Thus, by this comparative PET analysis there were 1,095 sites (45%) occupied uniquely in ESC (Figure 4A). Conversely, among the 857 high-confidence sites found in NSC, only ten were not also found in ESC. If the stringency of our cutoffs was raised to PET10+, then for ESC we found 153 sites (19%) that were not in enriched in NSC, and there were no sites enriched only in NSC. To assess the most appropriate threshold for a comparative analysis, the degrees of PET overlaps from the two libraries were cumulatively assessed at each cutoff from PET2+ to PET10+ (Figure 4B). For a threshold above PET5+ in the NSC, there was complete overlap with ESC PETs. The converse was not true: even at the most stringent cutoff, PET10+, only 80% of ESC PET10+ sites have an equivalent in NSC. qPCR carried out on randomly selected sites confirmed the common (19/19, 100%; Figure S11) and ESC-specific binding sites (13/20, 65%; Figure S12). Thus, the ChIP-PET results were consistent with the results obtained in our ChIP-chip experiments, which indicated that up to 30% of REST binding sites are selectively bound in ESC relative to NSC and that there were few, if any, sites exclusively bound in NSC.

To verify the pluripotency-specific nature of the ESC-specific PETs, we compared our data with a previous whole-genome study of REST binding in mouse kidney cells [22] (Figure 4C). Consistent with our hypothesis, we observed a robust and statistically significant lack of REST binding at ESC-specific loci in kidney cells. These ESC-specific PETs are unlikely to be some kind of statistical noise, since the majority contain full-length RE1 motifs, albeit at a lower rate than the commonly bound PET set (Figure 4C).

The ChIP results indicated that there were highly overlapping and yet distinct patterns of REST occupancy in ESC and NSC, two cell types that have unique developmental potential. REST is known to be a repressor of neuronal gene expression in non-neuronal cells and RE1 sites preferentially map to neuronally expressed genes [18,19,33]. Our data show that there are substantially more sites that bind REST than previously predicted, so we asked what the nearest potential target genes are among the expanded repertoire of binding sites. Full lists of target genes can be found in Dataset S4.

The expanded number of binding sites for REST in ESC led us to ask whether REST controls an ESC-specific repertoire of target genes. To test this, we compared the ESC-specific and ESC-NSC common target sets by gene ontology analysis [34]. Robust statistical filtering yielded several gene ontology terms that were significantly different in their association with the two gene sets. Gene categories relating to neuronal function and development were depleted among the ESC-specific set compared with the common genes, although it is important to note that such terms remain highly enriched in the ESC-specific set when compared with the set of all genes. In contrast, a number of ontology categories were significantly enriched in the ESC-specific set, even following Bonferroni correction; among these were genes mediating the Wnt signaling pathway, in addition to integrins, kinases and chromatin binding proteins (Figure 4D; see Dataset S6).

In addition to differential gene targeting, ESC-specific and ESC-NSC common PET clusters have distinct sequence properties: the former tend to exhibit weaker sequence conservation (Figure 4E). Common ESC/NSC clusters have higher quality RE1 motifs, as measured by their RE1 PSSM score (Figure 4F), resulting in elevated levels of in vivo REST recruitment (Figure 4G).

### Transcriptional Regulation of REST Target Genes in Stem Cells

**Figure 5:**
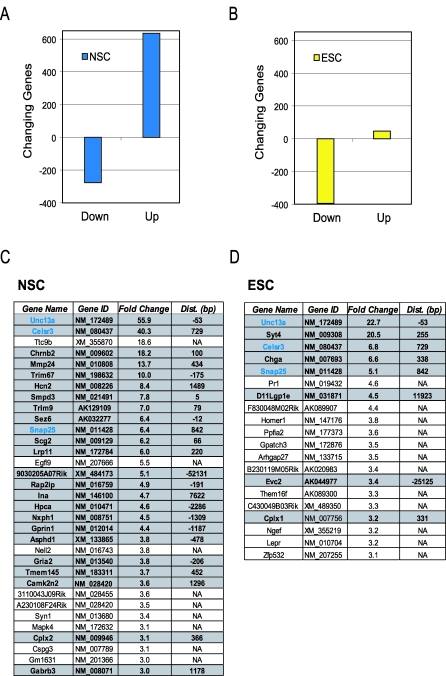
Diverse Outcomes of REST Function on ESC and NSC Gene Expression Profiles (A and B) The numbers of genes decreasing (“down”) and increasing (“up”) in response to DN:REST treatment are shown (*p* < 0.01 threshold for statistical significance) for (A) NSC and (B) ESC. (C and D) Genes with significant (*p* < 0.01) changes (≥3-fold) in expression were tabulated for (C) NSC and (D) ESC in response to DN:REST. Gray shaded genes are those with associated REST binding sites at the indicated distance (“Dist.”) from the TSS. Unshaded genes have no REST binding sites within 100 kbp. Common genes are printed in blue.

Our mapping of REST binding sites in ESC and NSC indicated that there were distinct patterns of occupancy in the two cell types. We next asked which genes are transcriptionally repressed by REST in these cells. To this end, we profiled gene expression in ESC and NSC in which the activity of REST was blocked by a dominant-negative form of REST (DN:REST). DN:REST comprises the eight zinc fingers of the REST DNA binding domain, but lacks the N and C termini; it thus derepresses transcription of REST target genes [28]. An adenovirus was used to efficiently deliver DN:REST to the NSC. After 48 h of REST derepression, global changes in gene expression were measured by DNA microarray analysis. We detected expression of ∼21,000 genes, of which 911 genes were significantly altered (*p* < 0.01) in NSC in the presence of DN:REST (Dataset S5). Overall, 635 (3.0%) and 276 (1.3%) of the expressed genes showed statistically significant up- and down-regulation, respectively (Figure 5A). Given that REST is a transcriptional repressor, it is anticipated that, in response to the inhibition by DN:REST, direct target genes would be up-regulated. Thus, down-regulated genes are likely to be downstream, indirect targets of REST. We asked whether the most differentially expressed genes had evidence for genomic occupancy by REST. The gene nearest to each REST binding site was identified. There were 33 genes with expression elevated 3-fold or greater, of which 24 (73%) had an associated binding site (Figure 5C). Given that this number is far lower than the total number of expressed genes associated with a REST PET cluster, these data indicated that only a small proportion of bound genes are actually derepressed by DN:REST in NSC. However, among the derepressed genes, those that were most responsive to REST knock-down did have an associated REST binding site.

We also investigated the transcriptional response of ESC to DN:REST over-expression. Unlike NSC, ESC cannot be infected by adenovirus, so instead they were transfected with a DN:REST construct and enriched by fluorescently activated cell sorting (FACS) to select for those strongly expressing DN:REST. After 48 h of DN:REST expression, gene expression profiling was carried out on these cells, showing that 441 genes were significantly differentially expressed in response to DN:REST: 395 (1.9%) were down-regulated, but only 46 (0.22%) were up-regulated (Figure 5B). The 20 most up-regulated genes (≥3-fold) show a strong bias for direct recruitment of REST: 40% are associated with a REST PET cluster (Figure 5D).

The distinct methodologies we used for DN:REST delivery preclude a rigorous comparison of transcriptional response in ESC and NSC. It is possible that different levels of DN:REST expression and temporal induction of expression lead to differential gene responses in the two cell systems. Of the 441 and 911 genes that responded to DN:REST in ESC and NSC, respectively, only 17 (1.3%) were similarly altered in both experiments, of which 11 were associated with a REST PET cluster. This was rather unexpected given that there was such a high concordance (80%) of REST sites occupied in ESC and NSC. Among the genes commonly elevated in ESC and NSC were *Celsr3*, *Snap25*, and *Unc13a*, which were occupied by REST and among the most highly induced in both cell types. *Unc13a* encodes a synaptic vesicle protein and was not a computationally predicted target of REST: its REST binding site, which we identified 53 bp upstream of the transcriptional start sites (TSS), does not closely match the canonical RE1 motif, though it does match the RE1 PSSM when similarity constraints are relaxed. Tandem canonical RE1 motifs were mapped in close proximity (<1 kb) to *Snap25*, a regulator of neural transmitter release [20], and *Cels3r*, a G-protein-coupled receptor, which plays a role in neuronal development. All the other most responsive genes had either a canonical or noncanonical RE1 motif.

**Figure 6:**
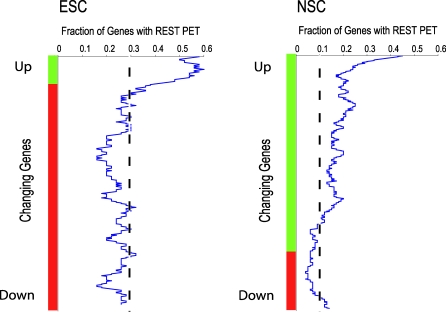
Recruitment of REST to Repressed Genes Significantly changing genes (*p* < 0.01) were ranked by their fold expression change in response to DN:REST (green, increased by DN:REST; red, decreased). For a sliding window, the fraction of genes within 100 kb of a REST PET cluster is plotted. The dashed line represents the mean value for all genes in the genome.

It was somewhat surprising that the vast majority of the genes with an associated REST binding site were not derepressed by DN:REST. We noted that the most up-regulated genes have sites in very close proximity to their TSS, often within 1 kb (Figure 5C and 5D). To investigate this further, we plotted the fold change in expression for each gene versus the distance of that gene to the nearest mapped RE1 site (Figure S13). This analysis clearly indicated a strong bias for the most differentially expressed genes to have an RE1 site proximal to its TSS. It is noteworthy that this bias for RE1 sites was associated only with the genes up-regulated, but not those down-regulated, by DN:REST, which is consistent with the expectation that REST acts as a repressor (Figure 6).

As expected, many of the genes that contain REST binding sites in NSC and showed elevated expression in response to DN:REST encode proteins that have neuronal functions, such as: neurotransmitter receptor subunits *Chrnb2*, *Gria2*, *Gabrb3*; neuronal adhesion-associated molecules *Ina*, *Cspg3*, *Nxph1*, *Mmp24*; and molecules associated with secretory functions *Chrnb2*, *Scg2*, *Trim9*, *Trim67*, *Cplx2*. This was expected as the NSC are poised to differentiate exclusively toward the neural lineage. In ESC, DN:REST also induced genes linked to neuronal function, in particular, synaptic vesicle biology: *Syt4*, *Snap25*, *Unc13a*, *Cplx1*, and *Chga*. These genes are also associated with neuroendocrine secretion, perhaps indicating a core requirement in both ESCs and NSCs to ensure active repression of gene products involved in vesicular secretion.

### REST Is a Part of the Oct4, Sox2, and Nanog Regulatory Network in ESC

**Figure 7:**
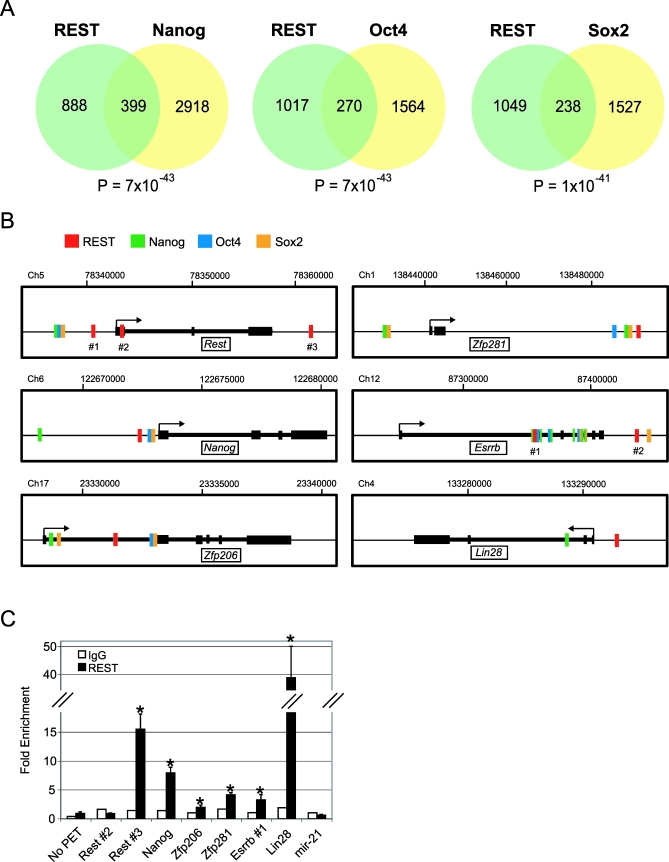
Integration of REST with the ESC Pluripotency Network (A) Venn diagrams show the overlap of target gene sets for REST and the indicated pluripotency transcription factors [35]. Statistical significance was calculated using the hypergeometric function. (B) REST is recruited to a number of pluripotency genes in ESC and coregulates them with the pluripotency transcription factors Nanog, Oct4 and Sox2. Thick lines represent gene exons, thinner lines introns. Colored blocks represent transcription factor recruitment in ESC validated by ChIP-PET (REST) or ChIP-sequencing (Nanog, Oct4, Sox2) [35]. (C) Conventional ChIP-qPCR was carried out on independent REST ChIP samples (*n* = 5) (black bars) to validate recruitment to pluripotency genes. In the case of mir-21, primer sequences were those described in Singh et al. [39]. Error bars represent standard error of the mean. Statistical significance was assessed by comparing data to a non-PET containing region (‘No PET'), using Student's *t*-test (* *p* < 0.05). No enrichment was detected using a control non-specific IgG (white bars).

REST had been implicated previously in the transcriptional regulatory networks that regulate ESC pluripotency, as the *Rest* gene is a target of Oct4, Sox2, and Nanog binding [2,5]. We explored more fully the connection between REST and the pluripotency transcription factors Oct4, Sox2, and Nanog. Recently our colleagues completed a detailed mapping of binding sites for Oct4, Sox2, and Nanog in ESC [35]. Very deep sequencing of ChIP DNA from ESC identified 1,834, 1,765, and 3,317 genes with binding sites for Oct4, Sox2, and Nanog, respectively, either within the gene itself or not more than 10 kb upstream of the target gene. These data confirm and extend earlier studies [2] in which these three transcription factors represent a core regulatory complex in ESC. By comparing the list of REST target genes with those of Nanog, Oct4, and Sox2, we found a statistically significant integration of target gene repertoires (Figure 7A). Of the 1,287 REST target genes, 270 (21%), 238 (18%), and 399 (31%) of these were also targets of Oct4, Sox2, and Nanog, respectively. In addition, 107 genes were targets of all four factors in ESC, including *Rest* itself (having three REST PET clusters), and several transcription factors implicated in ESC self renewal, such as *Nanog* [36,37] and *Zfp206* [5,23] (Figure 7B). We also noted that the gene of the reprogramming factor *Lin28* [38] contains a REST PET cluster in the proximal upstream region (Figure 7B). Using conventional ChIP-qPCR, we validated REST recruitment to *Nanog*, *Zfp206*, *Zfp281*, *Esrrb*, and *Lin28*, in addition to the *Rest* gene itself (Figure 7C). Furthermore, expression of *Zfp206*, *Zfp281*, and *Lin28* was induced by DN:REST (Figure S14). Surprisingly, we could find no evidence for recruitment of REST to the gene for the microRNA mir-21, as was reported recently [39] (Figure 7C). Oct4, Sox2, and Nanog have been shown to form an autoregulatory circuit where every factor regulates its own gene and that of the other two [5,40]. Our mapping data indicate that REST also forms such an autoregulatory circuit. These results support the hypothesis that REST is a component of the pluripotency network that includes Oct4, Sox2, and Nanog, which together control differentiation and pluripotency in ESC.

## Discussion

A central aim of biology is to reconstruct the transcriptional circuitries governing cell identity and differentiation during embryonic development. ESC and NSC lines have emerged as tractable and meaningful in vitro models in which to use high-throughput genomic techniques to map such circuitries. Given the wholesale and rapid changes in transcriptional activity that accompany differentiation, it is imperative to understand how transcriptional regulatory networks change during development. An obvious model, therefore, is a transcriptional regulator such as REST with important roles in multiple, related cell types such as ESC and NSC. In the present study, we mapped REST regulatory targets in ESC and NSC by complementary microarray and sequencing methods. We have shown that REST recruitment has dual components, consisting of an apparently cell-type–independent core population of binding sites in addition to a substantial pluripotency-associated set found only in ESC. The diversity of the REST regulatory network was even greater when analyzed on the transcriptional level, where we observed almost complete discordance in gene regulation in ESC and NSC. We have also expanded our understanding of REST's role in ESC by showing that it shares a substantial set of target genes, including *Rest* itself and *Nanog*, with Oct4, Sox2 and Nanog, the core pluripotency transcription factors.

In this genome-wide, comparative study, we found evidence for diversity in the REST recruitment profile between cells of various differentiation capabilities. The three-way comparison of recruitment in ESC, NSC, and fibroblasts demonstrated a substantial commonality in REST recruitment, in addition to a large minority of ESC-specific binding. However, even among the commonly bound loci, we observed large variation in the level of REST recruitment. Hierarchical clustering carried out on the data confirmed that, for this set of three cell types, the REST binding profile is most strongly influenced by pluripotency. In contrast, we could find no evidence for specific binding sites in either NSC or fibroblasts (Figures 2 and 4), suggesting that the REST recruitment profile is most extensive in ESC and decreases in line with loss of developmental potential.

These findings suggest that the unique genomic and chromatin organization of ESC [1] is also reflected in the recruitment profile of generic transcription factors such as REST. What is the mechanistic basis for this promiscuous recruitment in pluripotent cells? One possibility is that weaker RE1 motifs (with lower affinity) are only bound under the higher REST concentrations found in ESC [13]. This is supported by the fact that most of the ESC-specific sites have more degenerate RE1 motifs (Figure 4F). However, this interpretation may be overly simplistic: among the commonly bound sites, many were occupied to a much greater extent in NSC than in ESC (Figure 2). Furthermore, contrary to previous reports [13], we did not observe any difference in the total levels of REST protein in ESC and NSC (unpublished data). These data suggest that the difference in overall patterns of REST occupancy is not simply a consequence of REST protein concentrations. Given the profoundly different chromatin architecture observed in ESC [1,41], it is likely that many chromatin domains that are accessible to soluble factors in ESC become inaccessible at subsequent stages of differentiation. Another related possibility is that nucleosome positioning around RE1s is instructive for REST recruitment and serves to exclude REST from many sites upon loss of differentiation. Our recent demonstration that REST recruitment relies upon the ability of its cofactor, Brg1, to remodel local chromatin in an acetylation-dependent manner [42] lends weight to these arguments. Finally, other transcription factors may serve to recruit REST to weaker RE1s in an ESC-specific manner. This effect has previously been observed for the nuclear hormone receptor, ERα [43]. We are currently investigating all these possibilities by a combination of techniques used in this paper.

Transcription factor–target gene relationships have generally been inferred from ChIP evidence alone. In the present study, we avoided such assumptions by assimilating gene expression data with our PET analysis of REST recruitment. Specifically, we surveyed the functional response of all known genes to inhibition of REST by a dominant-negative construct. At the level of sensitivity of our assay at least, we found that only a small minority of detectable genes to which REST is recruited actually respond to its removal. This confirms, on a genome-wide level, previous observations that the removal of REST is often not sufficient for target gene derepression [12–14,44]. It is likely that, in such circumstances, gene activation requires the presence of particular activating transcription factors, or that repression by additional, REST-independent mechanisms must also be removed. Gene response is, however, strongly influenced by the relative location of REST binding sites to the TSS; specifically, TSS-proximal binding sites strongly repress gene transcription, from either upstream or downstream, and that the potency of this regulation drops rapidly within 2–3 kb (Figure S13). These data raise important new questions over the precise mechanisms governing gene regulation by REST. What factors determine whether a bound gene will respond to REST? Given the heterogeneity of responsiveness, we suggest that complex subnuclear organization determines which REST-bound loci have access to appropriate corepressor complexes, and therefore which genes are repressed. The data also lead us to question why the majority of high-quality RE1 motifs are at non-promoter loci [18] if they are broadly incapable of regulating gene transcription. In any case, these findings force us to consider that genome-wide mapping projects alone are insufficient for meaningful reconstruction of gene regulatory networks without accompanying functional data on gene expression.

To date, genomic surveys of REST target genes (including this study) have demonstrated their significant enrichment for genes relating to nervous system development and function [18–20,23]. Therefore, we were surprised to find that among ESC-specific REST targets, there are a large and significant number of genes encoding members of the Wnt signaling pathway (Figure 4D and Dataset S6). Wnt, a crucial determinant of both pluripotency and mesendodermal fate in ESC, is tightly controlled by both activating and repressive mechanisms in ESC [31,45,46]. Repression of Wnt and Wnt receptor genes is thus an important candidate mechanism by which REST maintains the pluripotent state. Repression of Wnt signaling by REST may also contribute to tumor suppression. REST was identified as a suppressor of human epithelial cell transformation and *REST* is frequently deleted in colorectal tumors [47]. Deregulated Wnt signaling is a frequent event in the genesis of many tumor types and plays an important role in the proper maintenance of the stem cell niche of the colonic epithelium [48]. In light of the connection we have established between REST and Wnt, it is possible that REST plays an important role in regulating Wnt signaling and that loss of REST function leads to tumor initiation. It will be interesting to determine whether REST is expressed in tissue stem cell populations and, if so, whether it regulates expression of Wnt pathway components. It currently remains unclear to what degree REST is responsible for regulating the Wnt pathway. It should be noted that the Wnt pathway genes were not among those derepressed by DN:REST in ESC. It seems likely that tight regulation of the Wnt pathway is critical and would, thus, be mediated by many competing, and reinforcing circuits that converge on this node in the transcriptional network.

In addition to the Wnt pathway, we found other strong evidence that REST is an important controller of pluripotency in ESC. A highly significant number of genes (200–400) are commonly targeted by REST and the pluripotency factors Oct4, Sox2, and Nanog (Figure 7); 107 genes are targets of all four factors. Many of these genes encode transcription factors, including Znf206, Esrrb, and Nanog, which have all been implicated in pluripotency maintenance. The reprogramming factor Lin28 is bound by REST and Nanog. The *Rest* gene itself is a common target of all four factors; thus, REST autoregulation would appear to be an evolutionarily conserved property, given its previous observation in human [49] and now in mouse. In response to recruitment of REST, we showed that *Zfp206*, *Zfp281*, and *Lin28* are transcriptionally repressed. Surprisingly, other pluripotency genes did not show changes in expression at the level of sensitivity of our assay. These data suggest that REST does repress the pluripotent phenotype at the level of transcription, but only weakly in cultured ESC. It is clear, however, that REST does prevent expression of many neural genes in ESC. We hypothesize that REST may be capable of repressing pluripotency genes such as *Nanog* at distinct developmental time points (which may not be faithfully represented by the ESC model), perhaps depending on the availability of correct corepressor molecules, which are known to change dynamically during ESC differentiation [5]. In this way, REST may be both a pro-pluripotency gene—by repressing neural phenotype in ESC—and an anti-pluripotency gene—by repressing pluripotency during subsequent differentiation. Finally, we could find no evidence for recruitment of REST to mir-21 in mouse ESC, as reported recently [39] (Figure 7C and Figure S15). Furthermore, mir-21 levels were unaffected by REST in our previous study [50], suggesting that the reported regulation of mir-21 may in fact be an indirect effect.

**Figure 8:**
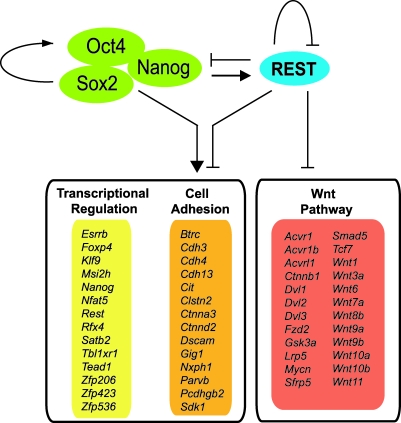
A Model of REST Regulation of Pluripotency and Differentiation in ESC Yellow and orange panels contain a selection of genes that are commonly targeted by REST, Nanog, Oct4 and Sox2. Red panel contains Wnt pathway-related genes (as identified by the Panther database) that are bound by REST. Activating (arrows) and repressive (bars) regulation is inferred from ChIP binding data.

Together, our results suggest a model in which the intersection of activating (Oct4, Sox2, Nanog) and repressive (REST) transcriptional signals control ESC pluripotency (Figure 8). The outcome of these opposing forces is expression levels of a large number of genes that are appropriate for the pluripotent state. In addition to potentially antagonizing pluripotency by binding genes such as *Nanog* and *Zfp206*, REST also appears to promote pluripotency through repression of multiple components of the Wnt pathway. The regulatory relationships suggested by our whole-genome mapping study will need to be functionally confirmed in future by knock-down of REST, which will lead to derepression of target gene expression; furthermore, it is possible that such regulation takes place only during stages of differentiation subsequent to that represented by the ESC as discussed above. Regardless of such details, however, our findings show that REST has a complex role in both promoting and antagonizing the pluripotent state.

## Materials and Methods

### Cell culture.

E14 cells (American Type Culture Collection) were cultured feeder-free as described [51]. NS5 neural stem cells were grown as described in [29]. NIH3T3 fibroblasts were cultured in Dulbecco's Modified Eagle's Medium supplemented with 10% fetal calf serum at 37 °C in 5% CO_2_. Details of ESC and NSC differentiation and immunohistochemistry can be found in the Text S1.

### Chromatin immunoprecipitation (ChIP).

ChIP was performed according to the Hinxton protocol [23]. Briefly, sonicated, cross-linked chromatin from 2 × 10^7^ cells was immunoprecipitated (IP) using 10 μg anti-REST antibody (Upstate 07–579). Immune complexes were collected using 50 μl of a 50% (v/v) slurry of BSA-blocked Protein G Sepharose. The same amount of nonspecific rabbit IgG was used in control IPs to gauge background, and non-IP Input DNA samples were also prepared for reference ChIP-chip hybridizations.

### Construction, hybridization, and scanning of RE1 ChIP-chip.

The RE1 ChIP-chip design was based on 1,319 RE1s from the mouse genome that had a PSSM score >0.90 [18]. Centered on each RE1, a 200-bp window was searched for appropriate 50mer hybridization probes with the following criteria: (1) 40–60% GC content; (2) no secondary structure; (3) ≥15 nt difference between all 50mer probes; (4) contiguous match between probes of ≤25 nt. 1,095 RE1s satisfied these criteria. Additionally, 92 negative control non-RE1 probes and two sets of tiled RE1-bearing promoters (*Nppa* and *Syt4*) were included in the design. Amine-conjugated DNA probes were synthesized and printed in duplicate onto Codelink Activated Slides (GE Healthcare).

Non-IP DNA (Input, 250 ng) and 46 μl ChIP DNA were amplified following the manufacturer's instructions (Bioprime (Exo-) kit, Invitrogen). Purified, amplified DNA (1 μg) was then labeled with Cy3 or Cy5 using a ULS arrayCGH labeling kit (Kreatech Biotechnology). Corresponding labeled DNAs were subsequently pooled, mixed with 90 μg mouse CoT-1 DNA (Invitrogen) and concentrated with Microcon YM-30 columns (Millipore) to a volume of 5 μl. The resultant concentrates were each mixed with 2 μl Kreablock (Kreatech Biotechnology), 80 μg yeast tRNA, 40 μg herring sperm DNA, 19 μl DIG Easy Hyb Buffer (Roche). The final 38-μl hybridization mixes were incubated for 15 min at 70 °C followed by 45 min at 37 °C, then hybridized to the microarrays (which were pre-hybridized with 60 μl DIG Easy Hyb Buffer at 42 °C for 1 h). Hybridizations were performed with a MAUI hybridization station (BioMicro Systems) at 42 °C for 20 h. Microarrays were washed (2x SSC+0.01% SDS at room temperature (RT) 5 min, 1x SSC at RT 5 min, 0.6x SSC at 60 °C 5 min, 0.2x SSC at RT 5 min), then scanned and imaged using a GenePix 4000B Scanner and software (Axon).

### ChIP-chip analysis.

We used a Gaussian Mixture Model to analyze raw ChIP-chip intensity data using the R package mclust [52]: the data are fitted to a finite number of Gaussian curves, each with a distinct mean and variance. See Text S1 for additional details.

### ChIP-PET analysis.

A total of ∼200 ng REST ChIP DNA, sheared to an average size of ∼750 bp, was used for the construction of each ChIP-PET library, essentially as described [31] and sequenced on a 454 Sequencer.

### Motif analysis.

We previously constructed a database of potential RE1 sites in the whole genome [18]. Comparing REST PET5+ clusters with this database identified 1,351 clusters in ESC that contained a candidate RE1 motif (Seqscan PSSM score >0.83). For the remaining 1,109 high-confidence clusters, we extracted 200 bp of flanking sequence and submitted them to the de novo motif-finding algorithms MEME [53] and Weeder [53]. These programs identified a degenerate RE1 motif consisting of positions 7–17 of the full-length motif, as well as the left (positions 1–9) and (positions 12–21) right half-sites of the canonical RE1 motif. This suggested that there were still weak canonical RE1 motifs present in the remaining clusters, as well as individual half-site motifs. To investigate this we scanned the 1,109 clusters using the full-length RE1 PSSM with a relaxed stringency threshold, as well as with PSSMs representing each of the left and right half-sites alone, and with both left and right PSSMs together (of *E* < 0.0001, using the technique described in [5]). For PET clusters containing both left and right motifs, we compiled their orientation as well as the distance separating the two motifs.

### Gene ontology.

Gene ontology analysis was carried out using the online package available at http://www.pantherdb.org [34]. Bonferroni-corrected *p*-values are shown as calculated by Panther based on binomial statistics.

### DN:REST expression.

The DN:REST construct [14], consisting of the REST DNA-binding domain alone, was cloned into the pCAG vector (Invitrogen) with a FLAG tag at the N terminus. 1 μg of pCAG_DN:REST (or empty pCAG vector) DNA was transfected with 2.5 μl Lipofectamine (Invitrogen) into E14. Transfection efficiency was 60–80%, as determined by the internal ribosomal entry site-driven green fluorescent protein (GFP) fluorescence. GFP-expressing cells were sorted by FACS and robust DN:REST expression was detected with the FLAG-antibody (Sigma-Aldrich). We used a recombinant adenovirus expressing DN:REST [14] for NS5 cells. The infection rate was 90–100%, as judged by GFP fluorescence. RNA was harvested after 48 h of DN:REST expression.

### RNA extraction and gene expression microarray analysis.

Total RNA was extracted from at least three biological replicates each of control cells and DN:REST-transfected (or infected) cells. RNA was labeled using a TotalPrep RNA Amplification kit (Ambion) and hybridized on Sentrix Mouse Ref-6 Expression BeadChip microarrays (Illumina) (see Text S1 for details).

## Supporting Information

Figure S1 Validation of ESC Pluripotency by Cell Sorting (208 KB AI).

Figure S2 Expression of Neural Stem Cell Markers by NS5 Cells (9.55 MB AI).

Figure S3 PCR Validation of ChIP-chip in ESC (237 KB AI).

Figure S4 qPCR Validation of ChIP-chip Data: Shared ESC/NSC RE1s (197 KB AI).

Figure S5 qPCR Validation of ChIP-chip Data: ESC-Specific Binding Sites (197 KB AI).

Figure S6 qPCR Validation of ChIP-chip Data: NSC-Specific RE1s (208 KB AI).

Figure S7 qPCR Validation of ChIP-chip Data: 3T3-Specific RE1s (197 KB AI).

Figure S8 qPCR Validation of ChIP-PET to Identify PET Cluster Size Cutoff (REST ChIP in ESC) (224 KB AI).

Figure S9 Comparison of ChIP-PET and ChIP-chip REST Binding Predictions (224 KB AI).

Figure S10 qPCR Validation of ChIP-chip Data: “No Motif” Binding Sites in ESC (200 KB AI).

Figure S11 qPCR Validation of ChIP-PET Data: Shared ESC/NSC RE1s (201 KB AI).

Figure S12 qPCR Validation of ChIP-PET Data: ESC-Specific Binding Sites (226 KB AI).

Figure S13 REST Represses Most Effectively from Promoter-Proximal Binding Sites (321 KB AI).

Figure S14 Regulation of Pluripotency Genes by REST (223 KB AI).

Figure S15 No Evidence for REST Recruitment to the Mouse mir-21 Locus (1.98 MB AI).

Dataset S1 ChIP-chip Data (432 KB XLS)

Dataset S2 ESC ChIP-PET Motifs (319 KB XLS)

Dataset S3 NSC ChIP-PET Motifs (125 KB XLS)

Dataset S4 ChIP-PET Target Genes (750 KB XLS)

Dataset S5 DN:REST Gene Expression Data (232 KB XLS)

Dataset S6 ESC-Specific Target Genes (21 KB XLS)

Text S1 Supplementary Methods (36 KB DOC)

## Abbreviations

ChIP: chromatin immunoprecipitation
ChIP-chip: chromatin immunoprecipitation microarray
DN:REST: dominant-negative REST construct
ESC: embryonic stem cell
FACS: fluorescently activated cell sorting
FDR: false discovery rate
GFP: green fluorescent protein
GO: gene ontology
IP: immunoprecipitate
NSC: neural stem cell
PET: paired-end tag
PSSM: position-specific scoring matrix
qPCR: quantitative polymerase chain reaction
RA: retinoic acid
REST: RE1-silencing transcription factor
RT: room temperature
SACO: serial analysis of chromatin occupancy
TSS: transcription start site
