# Supplementary material for: REST Regulates Distinct Transcriptional Networks in Embryonic and Neural Stem Cells
Source: PLoS Biol. 2008 Oct 28;6(10):e256. doi: 10.1371/journal.pbio.0060256 (PMC2573930; doi:10.1371/journal.pbio.0060256)
Supplement: Text S1 — (36 KB DOC) [file pbio.0060256.sd007.doc]

**Differentiation and Immunohistochemistry of E14 ESC**

For non-specific differentiation, E14 cells were cultured in the presence of 0.1μM retinoic acid (RA) for 8 days. Control cells were cultured in the absence of RA over the same time period.

Immunohistochemical staining of E14 cells was performed essentially described by Lim et al (*Mol Biol Cell*. 2007;18(4):1348-58) with the following primary antibodies (1:100): goat-anti-Nanog (W-18 SC30328, Santa Cruz Biotechnology, Inc, California, U.S.A.), goat-anti-Sox 2 (SC-17320, Santa Cruz Biotechnology, Inc, California, U.S.A.), monoclonal mouse-anti-SSEA1 (PHSB, Developmental Studies Hybridoma Bank, University of Iowa, U.S.A.), 1:200 dilution of mouse anti-Oct4 (SC5279, Santa Cruz Biotechnology, Inc, California, U.S.A.). This was followed by the appropriate secondary antibodies detecting mouse or goat IgG Alexa-Fluor 488 (Molecular Probes Inc, Oregon, U.S.A.) (1:500). Images were captured with a confocal microscope at 63 times magnification (LSM 510 Meta, Zeiss, Oberkochen, Germany).

**Differentiation and Immunohistochemisty of NS5 NSC**

Tripotentential differentiation of NS5 cells was performed essentially as described by Glaser et al. (*PLoS ONE*, 2007;2(3):e298). Briefly, cells were plated onto polyornithine/laminin coated coverslips. Cells were first grown in DMEM:F12 supplemented with L-glutamine, modified N2, 10ng/ml FGF2, 10ng/ml PDGFAA and 10μM forskolin for 4-5 days followed by withdrawal of growth factors and addition of 200μM ascorbic acid and 30ng/ml T3 (3,3',5 triiodo-L-thyronine) for a further 3-4 days.

Cells were then fixed in 4% paraformaldehyde before processing for immunofluorescence labelling. Antibodies used were: Primary - anti-O4 (mouse IgM, 1:200, Sigma), TuJ1 (mouse IgG2a, 1:1000, Covance) and anti-GFAP (mouse IgG1, 1:200, Millipore); Secondary – goat anti-mouse IgM Alexa-594 (1:500, Molecular Probes), goat anti-mouse IgG2a Alexa-488 (1:500, Molecular Probes) and goat anti-mouse IgG1 Biotin (1:100, Southern Biotech); Biotinylated antibodies were visualised using streptavidin-Alexa-350 (1:100, Molecular Probes). O4 labelling was performed before cell permeabilisation with 0.1% TX-100. All other antibody steps were performed following permeabilisation. Cells were mounted in Prolong Gold anti-fade mounting medium (Molecular Probes) and imaged using a Zeiss AxioImager Z1 and AxioCam MR3 with AxioVision software (Carl Zeiss). Images were assembled using Adobe Photoshop CS3. All images were taken using 63x/1.4 objective.

**ChIP-chip**

The Gaussian Mixture Model (GMM) approach is particularly suited to handling a biased microarray such as the RE1 ChIP-chip, since around 60% of the probes are enriched for REST binding (conventional analysis based on global normalization would unnaturally reduce the resulting enrichment ratios). Instead of global normalization, we normalized spot intensity ratios to the reference, REST-negative loci on the microarray. The median red:green ratio for each of the 184 (2x92) negative control probes was calculated. These values were transformed to have identical mean and variance as the lowest positioned Gaussian of the GMM fitting of the remaining probes. The probability of enrichment was calculated for every RE1 using a Single Array Error Model (SAEM), combining the data from five biological replicate experiments. RE1s were ranked by resultant P-value.

The false discovery rate (*FDR_K_*) at a position K of the ranked RE1s was estimated using the following formula:

$$FDR_{K}=\frac{\Pi_{0}Nf_{K}}{K}$$

Π_0_ is the proportion of non-enriched probes on the microarray (40% in ESC cells). It is estimated by performing an additional GMM analysis on the effective log fold-enrichment ratios from SAEM analysis. *N* is number of probes on the microarray (2896). *f_K_* is the false positive rate at position K: the ratio of the number of negative control probes, having P-value ≤ the K^th^ ranked site, to the total number of negative control probes (184).

Based on the ChIP-chip analysis we found 521 RE1 target sites which were commonly bound with a 10% FDR in ESC, NSC and NIH3T3 cells. The fold-enrichment ratio of each probe in each cell line was analyzed using TM4’s Multiexperiment Viewer (MeV) (60). Gene and sample trees were hierarchically clustered using Euclidean distance matrix and average linkage parameters.

**Illumina Gene Expression Microarray**

Beadstudio software (Illumina) was used to prepare background normalized data before importing into GeneSpring GX 7.3.1 analysis software (Agilent). The data was then subjected to data transformation/normalization by selecting the following options: (a) Data Transformation: Set measurements less than 10.0 to 10.0; (b) Per Chip: Normalize to 50th percentile; (c) Per Gene: Normalize to median. Gene filtration was subsequently applied on the dataset by retaining only genes with consistent ≥0.8 ‘Detection’ level (confidence level as defined by Illumina Beadstudio software) in 3 out of total 6 samples in each experiment. To identify genes with differential expression, Welch T-test was performed to compare transfectants to control, setting p-value cut-off (Benjamini and Hochberg False Discovery Rate) at 0.01.
